# IUC26562-86 MERIT AWARD: Integrating Transcriptomic Profiles and Clinical Outcomes Across MEET-URO Risk Groups in Metastatic Renal Cell Carcinoma: A Comparative Analysis with the IMDC Framework

**DOI:** 10.1093/oncolo/oyag286.019

**Published:** 2026-08-21

**Authors:** G Neola, N Salgia, A Moradi, S E Rebuzzi, P Rescigno, S K Pal, G L Banna

**Affiliations:** Portsmouth University Hospital NHS Trust - United Kingdom; City of Hope Comprehensive Cancer Center, Duarte, CA, USA - United States; City of Hope Comprehensive Cancer Center, Duarte, CA, USA - United States; Medical Oncology Unit 2, Ospedale Molinette, Azienda Ospedaliero-Universitaria Città della Salute e della Scienza di Torino, Torino, Italy - Italy; Translational and Clinical Research Institute, Newcastle University Centre for Cancer, Newcastle Upon Tyne, United Kingdom - United Kingdom; City of Hope Comprehensive Cancer Center, Duarte, CA, USA - United States; Portsmouth University Hospitals NHS Trust, Portsmouth, UK - United Kingdom

## Abstract

**Background:**

The MEET-URO scoring system is a validated prognostic tool in metastatic renal cell carcinoma (mRCC), but its biological correlates and comparative utility against the established IMDC framework require further elucidation.

**Methods:**

We analysed an institutional dataset from the City of Hope Comprehensive Cancer Centre, Duarte, CA, USA, comprising 86 patients with mRCC. Gene expression profiles from tissue samples were evaluated using ssGSEA to assign patients to the seven IMmotion151 non-negative matrix factorization (NMF) clusters and the three aggregated OPTIC-RCC phenotypic groups (Angiogenic/Immunological/Mesenchymal). Cellular deconvolution was performed using xCell. Clinical outcomes, including objective response rates (ORR) to first-line immune-oncology (IO)-based regimens versus targeted tyrosine kinase inhibitor therapies (TT), were stratified by both MEET-URO groups (Group 1-5) and IMDC risk classes (Favourable-Intermediate/Poor).

**Results:**

The cohort spanned MEET-URO Group 1 (n  =  19), Group 2 (n  =  25), Group 3 (n  =  27), Group 4 (n  =  12), and Group 5 (n  =  3). Cross-classification with IMDC revealed strong alignment: 89.5% of MEET-URO Group 1 patients were IMDC Favourable, whereas 100% of Group 4 and 5 patients were IMDC Intermediate/Poor. Significant biological differences emerged across the MEET-URO groups. Group 1 displayed prominent angiogenic and stroma-centric profiles, correlating with the OPTIC Group A phenotype (IO-VEGF-susceptible, NMF clusters 1 and 2). Conversely, Groups 4 and 5 exhibited significant enrichment in myeloid inflammatory pathways (p  =  0.036) and complex immunological networks, including T-cell CD4 Th2 infiltration (p  =  0.005) and higher overall immune scores (p  =  0.044). Therapeutic responses mirrored these genomic signatures. In MEET-URO Group 1, TT achieved an optimal ORR profile (5 responders vs. 2 non-responders). In contrast, Groups 4 and 5 showed a clear preference for immunomanipulation, with IO-based therapies demonstrating superior response dynamics (Group 4 IO: 6 responders vs. 2 non-responders; Group 5 IO: 2 responders vs. 0 non-responders). For intermediate cohorts (Groups 2 and 3), IO-VEGF combinations remained the pragmatic baseline.

**Conclusions:**

The MEET-URO scoring system closely mirrors underlying transcriptomic phenotypes and distinct microenvironmental configurations in mRCC. While aligning with the traditional IMDC risk stratification, the MEET-URO score provides biological granularity - differentiating angiogenesis-driven, TT-susceptible tumours (Group 1) from myeloid-inflamed, highly immunogenic, IO-responsive disease (Groups 4 and 5) - thereby serving as a potential driver for precision therapeutic selection.

